# Bayesian Classification and Regression Trees for Predicting Incidence of Cryptosporidiosis

**DOI:** 10.1371/journal.pone.0023903

**Published:** 2011-08-31

**Authors:** Wenbiao Hu, Rebecca A. O'Leary, Kerrie Mengersen, Samantha Low Choy

**Affiliations:** 1 Mathematical Sciences, Queensland University of Technology, Brisbane, Queensland, Australia; 2 School of Population Health, University of Queensland, Brisbane, Australia; 3 Australian Institute of Marine Science, The Oceans Institute, University of Western Australia, Crawley, Western Australia, Australia; 4 Biosecurity Statistics, Cooperative Research Centre for National Plant Biosecurity, Canberra, Australian Capital Territory, Australia; Genentech Inc., United States of America

## Abstract

**Background:**

Classification and regression tree (CART) models are tree-based exploratory data analysis methods which have been shown to be very useful in identifying and estimating complex hierarchical relationships in ecological and medical contexts. In this paper, a Bayesian CART model is described and applied to the problem of modelling the cryptosporidiosis infection in Queensland, Australia.

**Methodology/Principal Findings:**

We compared the results of a Bayesian CART model with those obtained using a Bayesian spatial conditional autoregressive (CAR) model. Overall, the analyses indicated that the nature and magnitude of the effect estimates were similar for the two methods in this study, but the CART model more easily accommodated higher order interaction effects.

**Conclusions/Significance:**

A Bayesian CART model for identification and estimation of the spatial distribution of disease risk is useful in monitoring and assessment of infectious diseases prevention and control.

## Introduction

*Cryptosporidium* causes gastrointestinal infection in humans and animals and is now the most common protozoan parasite associated with gastroenteritis [1]. Cryptosporidiosis diseases are sensitive to weather variability as temperature and/or rainfall can influence the development and transmissibility of *cryptosporidium* and may also affect people's health-related behaviour. However, there are complex spatio-temporal interactions between the potential explanatory variables of these diseases that motivate further investigation.

Spatial dependence and heterogeneity are well known as major features of in spatial analysis of disease risk [2], [3]. Spatial dependence can arise from the delineation of spatial units of observation (such as suburbs, statistical local areas and counties), spatial aggregation, and the presence of spatial exploratory factors. Spatial heterogeneity is related to the lack of stability over space of the spatial relationships between the observations [4], [5].

Bayesian methods have been shown to account more sensibly and comprehensively for uncertainty in inference than frequentist methods, particularly with regard to the handling of parameter and model uncertainty [6], [7], [8]. Bayesian algorithms such as Markov Chain Monte Carlo (MCMC) have allowed for more widespread application of Bayesian methods to many fields of scientific investigation, including public health [9].

Bayesian spatial conditional autoregressive (CAR) models are increasingly being used to estimate spatial variation in disease risk between spatially aggregated units [2], [10], [11]. These models are typically represented as a linear regression between the response and explanatory variables with additional terms to explain spatial correlation. These models thus incorporate and estimate spatial correlation while simultaneously estimating covariate effects. Recently, Bayesian spatial and spatiotemporal models have been used to study the geographical distribution of tropical diseases including Ross River virus, malaria and schistosomiasis [2], [12], [13], [14].

Classification and regression tree (CART) models provide an alternative representation of the relationship between a response variable and potential explanatory variables. These models have been shown to be very useful in identifying and estimating complex hierarchical (high order nonlinear interaction effect) relationships in ecological and medical contexts [15], [16], [17], [18]. CART models are accepted in many fields of research because they are easy to interpret, more flexible than conventional parametric regression models and have a good predictive power [16]. Bayesian CART models have also been developed [19], [20] but have yet to be widely applied [21], [22], [23].

In a previous study we used a frequentist CART model to assess the relationship between social-ecological factors and cryptosporidiosis [24]. In this study we apply the Bayesian CART algorithm developed by O'Leary [22] to predict the spatial distribution of the cryptosporidiosis infection using selected social-ecological factors and climate variables. We also compare the outcomes of the spatial CART model with those of the Bayesian spatial CAR model.

## Materials and Methods

### Data collection

The dataset considered here has been described elsewhere [24]. Briefly, we obtained the computerised dataset on notified cryptosporidiosis cases by local government areas (LGAs) in Queensland for the period of 1^st^ January–31^st^ December 2001 from the Queensland Department of Health. The dataset includes the onset date and place of onset of the notified cases of cryptosporidiosis infection, age and sex of the patients and laboratory test date. Weather (daily temperature and daily rainfall) and socio-economic index for areas (SEIFA) data were obtained for the same period from the Australian Bureau of Meteorology and the Australian Bureau of Statistics, respectively.

### Bayesian CART model

CART models are binary decision trees that are built by dividing the predictor space repeatedly into partitions, or nodes, based on splitting rules of the predictor variables [15]. The aim of partitioning the space in this manner is to progressively increase the homogeneity of the response variable *y* within each node. The response variable determines the type of tree and the homogeneity of the terminal nodes. If the response variable is categorical then a classification tree is used to predict the classes of the response, and assessment of homogeneity is based on (correct) allocation of observations within a node to a single class; alternatively if the response is continuous then a regression tree predicts the average response within a node, and assessment of homogeneity is based on the corresponding variance, deviance, residual sums of squares or similar measure.

This modelling approach facilitates the fitting of complex nonlinear interactions, such as combination of environmental and sociological variables to help explain spatial patterns of a disease (e.g. [8]), combinations of habitat variables describing ecological niches [11], or gene-gene interactions that explain diseases [25].

**Figure 1:**
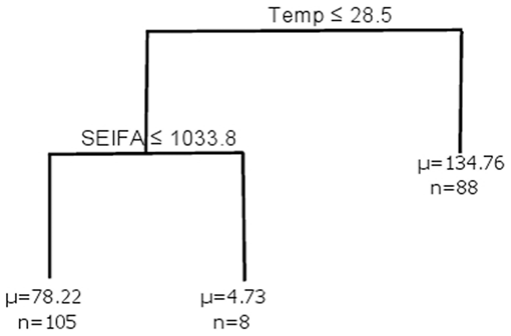
The best tree identified from Bayesian regression trees. At each terminal node the mean (μ) and number of individuals (n) are displayed.

Consider a response variable *y_i_* and predictor variables x_i*l*_, *i* = 1,…, *n*; *l* = 1,…, *L*. The partition of the response variable starts at the root node and divides the predictor space (observations *i*) at each internal or split node *S_k_*, *k* = 1,…, *K*−1, where *K* is the size of the tree (defined as the number of terminal nodes). At each splitting node *S_k_*, the partition is based on a splitting rule *R_k_*, of a variable *V_k_* and divides the observations {*y_i_*; *y_i_* 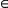 *S_k_*} into the left and right child node. Terminal nodes *T*_1_, …, *T_K_*, also called leaves, are the final nodes in which the predictor space is not split any further. At each splitting node *S_k_*, the *l*th predictor is selected as the splitting variable *V_k_* from the list of possible predictor variables *x_l_*. If this predictor is continuous, e.g. *S_1_* in Figure 1, then the splitting rule *R_k_* is based on a value *a* so *R_k_* = *a*, where min(*V_k_*)≤*a*≤max(*V_k_*). For example, at *S_1_* in Figure 1, *V*_1_ is Temperature and *R*_1_ is Temperature ≤32.5, so that observations with temperature less than or equal to 32.5 are partitioned to the left of the tree and the remainder are partitioned to the right. Alternatively, for a categorical response, *R_k_* is based on a class subset *c* so *R_k_* = *c*, where *c* 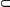 {possible levels of *V_k_*}. Letting ψ*_k_* represent the parameters corresponding to the assumed distribution of the data in the *k*th terminal node, the parameter vector θ*_k_* = (R*_k_*, S*_k_*, V*_k_*, ψ*_k_*) defines the parameter set or tree structure in this node; thus θ*_K_* = {θ*_k_*, *k = *1, … *K*}.

Following O'Leary [22], in a Bayesian framework, the joint distribution of the model parameters (size of tree *K*, tree structure θ*_k_* and response variable *y*) is modelled by
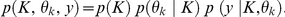
Here *p*(*K*) is the prior probability distribution for each model (where the model is defined by the number of terminal nodes *K*), *p*(θ*_k_|K*) is the prior probability distribution of the parameter set θ*_k_* given model *K*, and *p*(*y*|*K*, θ*_k_*) is the likelihood of the data *y* given the model *K* and the corresponding parameter set θ*_k_*. Bayesian analysis about the tree size *K* and tree structure θ*_k_* is calculated from the joint posterior distribution *p*(*K*, θ*_k_*|*y*).

For regression trees, if the (continuous) response variable *y* is assumed to have a normal distribution, then ψ*_k_* = (μ*_k_*, σ*^2^_k_*) and the likelihood is
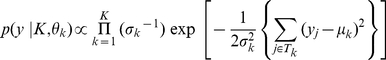
For classification trees, the (categorical) response variable *y* is typically assumed to have a multinominal distribution, so that if there are N categories, ψ*_k_* = (*p_k1_,..,p_kN_*) and the likelihood is
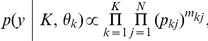
where *m_kj_* is the number of data points *y* at the *k*th terminal node *k* which are classified into the *j*th category.

The prior for the model is *p*(θ*_k_|K*) *p*(*K*), so that
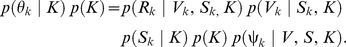
For a regression tree with a normal likelihood, a noninformative prior for *p*(ψ*_k_*|*V*, *S*, *K*) can be represented by a normal prior with a large variance for μ*_k_* and a uniform prior with a large range for σ*_k_*. For a classification tree with a multinomial likelihood, a noninformative prior for *p*(ψ*_k_*|*V*, *S*, *K*) can be represented by a Dirichlet prior for *p_k_* with hyperparameters equal to 1.

Dirichlet priors may also be used in both regression and classification trees for the splitting node *p*(*S_k_* | *K*), variables *p*(*V_k_* | *S_k_*, *K*), and splitting rules *p*(*R_k_* | *V_k_*, *S_k_*, *K*):
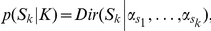


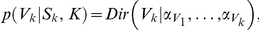


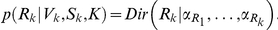
When no prior information is available about these variables, non-informative uniform distributions can be defined by setting all hyperparameters to 1, so that 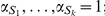


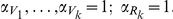



The prior on the size of the tree *p*(*K*) is assumed to be a truncated Poisson distribution with parameter λ (expected number of nodes in the tree),
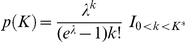



This prior imposes a left limit of *k>*0 because the minimum model contains one terminal node. The value of λ represents the expected number of splitting nodes is restricted to an interpretable size K^*^. In the case study considered here, this was taken to be λ = 10 [20].

In the present case study there was no information available about the model variables, so, noninformative priors were adopted. In other situations, if such information is available, then informed priors may be used instead. For example, in an analysis of habitat suitability of a threatened species, O'Leary et al. [23] discuss how to elicit from an expert the size of the tree, the relative importance of the variables, and the splitting rules for the most important variables. They also show how to translate this information into priors and combine with the data for Bayesian classification trees.

The sensitivity of the Bayesian CART model to the choice of priors has been investigated by O'Leary [22] for classification trees. The sensitivity analysis involved the investigation of the hyperparameters of the priors for tree size (number of terminal nodes), splitting nodes, splitting variables and splitting rules. The results indicated that the posterior distribution is relatively robust to these priors except for extreme choices of the hyperparameters.

The Bayesian CART models were fitted using the approaches suggested by Chipman *et al.* [19] and Denison *et al.* [20]. A reversible jump MCMC algorithm was used [20], [26], with single long chain [20]. The final stopping rule was based on the stability of the posterior distribution [20].

A fully Bayesian simulation from the posterior distribution could have been implemented via a greedy search algorithm. However, currently this is computationally infeasible because the parameter space is large and has an inflexible hierarchical structure. Instead we chose to follow the overall approach of Denison et al. (1998) and Chipman et al. (1998), by constraining the search algorithm to examine only the more optimal portions of the model space [19], [20]. This stochastic search algorithm is based on careful choice of model performance criterion to ensure that a range of good models are selected [22]. Therefore, Bayesian CART search algorithm produces a large number of trees, whilst traditional CART only produces one tree. The selection of the best classification tree, in Bayesian CART algorithm, is based on the research aim, in this case study the tree with the highest sensitivity and specificity.

Following O'Leary [22], the goodness of fit of a classification tree is assessed by several accuracy measures, calculated from the confusion or loss matrix (Table 1). The “best” tree can be defined as the one that minimizes/maximises one or more accuracy measures, depending on the aims of the study. In this paper the following accuracy measures were chosen: the misclassification rate (MCR) = (number of false positives (b)+number of false negatives (c))/total number (N), sensitivity = number of true positives (a)/(number of true positives (a)+number of false negatives (c)) and specificity = number of true negatives (d)/(number of true negatives (d)+number of false positives (b)). A set *S_G_* of *G* “good” trees is identified based on preset criteria, in this case study trees with highest sensitivity and specificity, and lowest MCR. The variables and splitting rules at each splitting node of the trees in *S_G_* are examined, and convergence is declared when the membership of *S_G_* and structure of the component trees has stabilised, i.e. the same trees are in the set *S_G_*.

**Table 1:** Confusion or loss matrix – classification of observed versus predicted presence (‘Yes’) and absences (‘No’) from Bayesian CART model.

Predicted	Observed		Total
	Yes	No	
Yes	a (true)	b (false)	a+b
No	c (false)	d (true)	c+d
Total	a+c	b+d	N

For each tree in the set of good classification trees *SC_G_* the following summary statistics can be examined: tree structure (variables, splitting rules and number of terminal nodes), sensitivity, specificity, deviance (−2×log likelihood *p*(*y|K*, θ*_k_*)), log likelihood and log posterior probability. From this set of good classification trees, depending on the aims of the analysis, a small number of trees may be chosen as the “best” trees, based on the modal tree structure (same size tree with the same variables and splitting rules), highest sensitivity and specificity, lowest deviance, and the highest likelihood and posterior probability.

For regression trees, the stopping criterion is based on posterior probabilities, deviance and residual sums of squares (RSS)
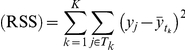
Therefore a set of *SR_G_ G* good *R* regression trees, for a certain number of iterations after burn-in, is identified to have the smallest RSS, minimum deviance and maximum likelihood and posterior probabilities of *p*(ψ*_k_*|*V*, *S*, *K*) (i.e. distribution of the data given the tree structure). Similar to classification trees, tree structure (variables, splitting rules and number of terminal nodes) in *SR_G_* is investigated. Once the membership of *SR_G_* and structure of the component trees has stabilised, this set of regression trees is declared “good”.

Bayesian models focus on the estimation of the model parameters (and model) conditional on all of the observed data. Overfitting of the Bayesian CART model can be assessed in the following manner. Following the practice adopted in cross-validation, the data can be split into a training and test dataset, using a stratified random sample to ensure equivalent allocation of presences and absences (for a classification tree) or subgroups (for a regression tree) [27], [28]. The model is then fit to the training dataset and the set of best trees is identified. For each tree, the posterior predictive distribution [27] is computed for both the training dataset and the test dataset and a confusion matrix based on the posterior predictive distribution and the observed data is computed. This is performed for each iteration of the MCMC algorithm, thus incorporating the uncertainty of the model parameters and the data in the evaluation. Finally, overfitting is assessed by comparing the accuracy measures (classification trees) or RSS (regression trees) between the training and validation datasets for the best trees. This approach is an adaptation of the typical use of predictive posterior distributions [27], in that instead of comparing the distribution of the observed data with that of future observations ỹ under a proposed model, here we compare these distributions of observations in the training and validation datasets.

The cryptosporidiosis dataset contains a large number of zero incidence rates (n = 1131 out of 1332 observations). To accommodate this, two Bayesian CART models were applied to incidence of cryptosporidiosis in LGAs: 1) a Bayesian classification tree in which the response is binary: presence/absence of cryptosporidosis; 2) a Bayesian regression tree in which the response is continuous: positive incidences rates, i.e ignoring zeros. This two stage approach is similar to hurdle and zero-inflated models [29].

### Bayesian CAR model

An initial descriptive analysis of cryptosporidiosis was performed. Crude standardised morbidity ratios (SMRs) for each LGA for the whole study period were calculated using standard methods [9], where SMR = (the observed number of cryptosporidiosis cases)/(the expected number of cryptosporidiosis cases). This model assumed that the observed counts of cases (*O_kt_*) for the *k*th LGA (*k* = 1…125) in the *t*th month in 2001 follow a Poisson distribution with mean (*μ*_kt_), that is,
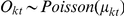
and
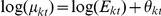


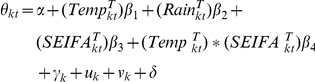
where *α* is the intercept, *β_1_* is the coefficient for temperature, *β_2_* is the coefficient for rainfall, *β*_3_ is the coefficient for SEIFA, *β_4_* is the interaction coefficient of temperature and SEFIA, γ is a LGA-level temporal trend coefficients, *u* is LGA-level variation that is spatially structured (ie. spatially-structured factors not explained by the model covariates), *v* is spatially unstructured LGA-level variation, and *δ* is the amplitude of seasonal oscillation in the month-specific random effects, which was modelled by a sinusoidal term *cosine*(2π×t/12). Spatial correlation between LGAs was modelled using a CAR prior for *u*, using a simple adjacency weights matrix [9].

Parameter estimation was obtained via MCMC simulation using an initial burn-in of 5000 iterations and subsequent set 100,000 interactions for estimation. Convergence was assessed by examining posterior density plots, history plots and autocorrelation of selected parameters. Model selection was performed using the deviance information criterion (DIC), where a lower DIC suggests a better trade-off between model fit and parsimony. Poisson regression models were developed in a Bayesian framework, using the WinBUGS software version 1.4 [30].

## Results

**Figure 2:**
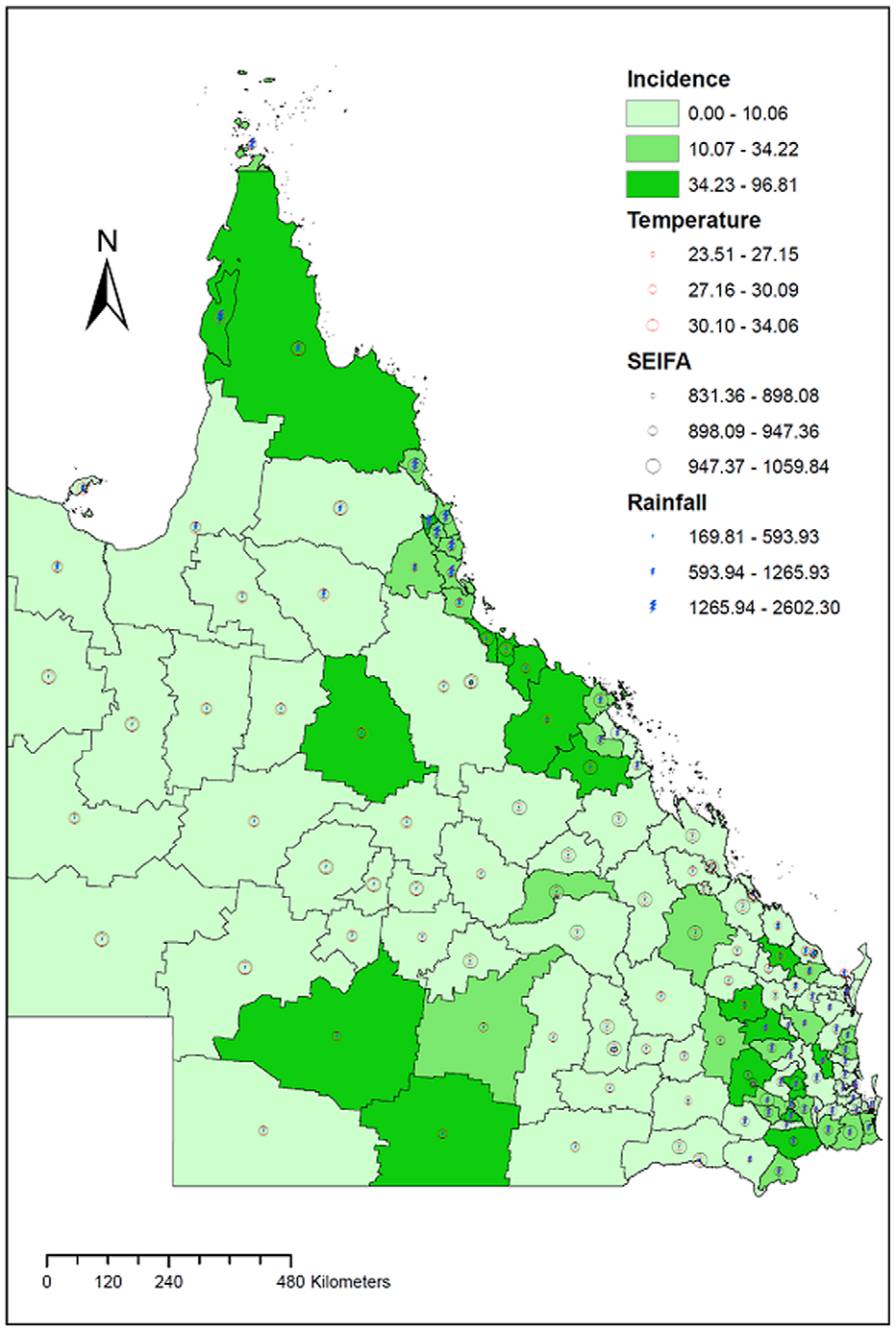
The observed spatial distribution of SEIFA, temperature, rainfall and annual average incidence rates of cryptosporidiosis.

Figure 2 shows the spatial patterns of cryptosporidiosis, rainfall, temperature and SEIFA in Queensland by LGA. The figure confirms that all these variables varied with geographical location.

### Bayesian classification tree

**Figure 3:**
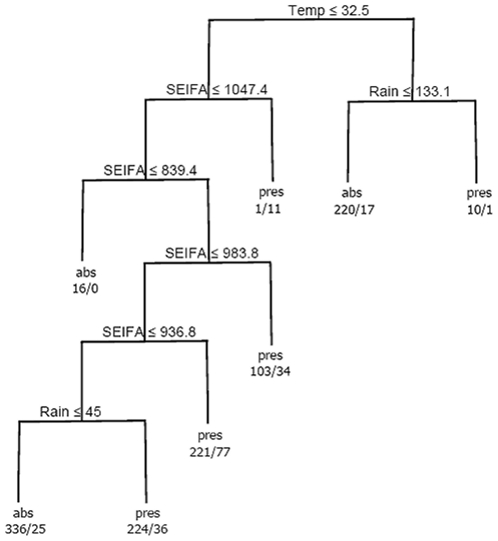
The best tree identified from Bayesian classification trees. At each terminal node the predicted category of presence or absence is denoted respectively by pres or abs. The two numbers directly below this are in general a/b (e.g. 16/0) which denotes the number of observed absences “a” and presences “b” that are classified into this particular node.

A set of five good Bayesian classification trees, with the highest sensitivity, specificity and lowest deviance, are displayed in Table 2. The first tree has the highest sensitivity and specificity, and lowest deviance. Since the focus of this case study was on correct prediction of presence (highest sensitivity) the first tree was selected as the best. This tree, depicted in Figure 3, indicates that presence of cryptosporidiosis was predominantly explained by a high-order nonlinear interaction between temperature, SEIFA and rainfall. The probability of cryptosporidiosis was largest when temperature was high and rainfall was low, temperature was low and SEIFA was very low, and temperature was low and SEIFA was mid-range but rainfall was low.

**Table 2:** Top 5 of the set of 16 best trees (based on sensitivity, specificity, accuracy and deviance) for Bayesian classification trees.

	Training dataset				Validation dataset				
Trees	Sens	Spec	Post	Dev	Sens	Spec	Post	Dev	Size
1	0.776	0.527	−406.08	807.78	0.825	0.513	−93.94	183.51	8
2	0.783	0.502	−405.74	807.32	0.825	0.491	−93.65	183.15	9
3	0.789	0.501	−420.28	836.20	0.800	0.496	−100.59	196.82	8
4	0.783	0.538	−417.91	831.44	0.775	0.531	−103.44	202.52	8
5	0.783	0.517	−409.40	814.44	0.750	0.482	−101.63	198.92	11

The table displays sensitivity (Sens), specificity (Spec), posterior (Post) and deviance (Dev) for both the training and validation datasets. The size of the tree (K; number of terminal nodes) is also shown.

Table 3 shows the quantiles of sensitivity, specificity and log posterior (distribution of data given the tree structure) for training and validation datasets over all accepted classification trees. This shows that the Bayesian CART algorithm search space includes trees with very low (close to zero) to very high (close to one) sensitivity and specificity.

**Table 3:** Quantiles of sensitivity, specificity and log posterior for training and validation datasets over all accepted trees, for Bayesian classification trees.

		2.50%	50%	97.50%
Training	Sensitivity	0.081	0.466	0.938
	Specificity	0.108	0.638	0.976
	Log posterior	−441.580	−414.580	−394.710
Validation	Sensitivity	0.050	0.475	0.950
	Specificity	0.124	0.646	0.987
	Log posterior	−109.860	−100.090	−91.965

Overfitting of Bayesian classification trees was explored by investigating the quantiles of sensitivity and specificity for training and validation dataset, over all accepted trees. Table 3 reveals similar 95% CIs for sensitivity and specificity between the training and validation datasets, indicating no over-fitting. However, for the validation dataset, the fourth and fifth trees have slightly higher sensitivity than the first tree.

### Bayesian regression tree

The Bayesian CART algorithm was applied to positive incidence rates of cryptosporidium. The set of five best regression trees (with lowest RSS and deviance) have the same log RSS (−58.96 and −58.47), log posterior (−16.18 and −13.56) and deviance (22.58 and 17.35) for both training and validation dataset respectively. The only difference between these trees is the splitting rules, which have all resulted in the same *y* observations being classified into the same terminal nodes. Over the 300,000 iterations, the iteration number for each of these five trees are very different, indicating that the Bayesian CART did not get trapped in local maxima. The first and second trees were designated as the ‘best trees’ since they were most consistently accepted in the set of good trees.

The best regression tree modeling positive incidence rates of cryptosporidium is displayed in Figure 1. There are three groups of positive incidence rates of cryptosporidium, ranging from low to high incidence. A monthly mean incidence rate of cryptosporidium of 78.22/100,000 (n = 105; far left terminal node) occurs in areas with temperatures less than or equal to 28.5° and SEIFA less than or equal to 1033.8. The monthly mean incidence rate is reduced to 4.73/100,000 when temperatures are the same but SEIFA is greater than 1033.8. The highest monthly mean incidence rate (134.76/100,000) occurs when the temperature is greater than 28.5°.

The quantiles of log RSS, deviance and log posterior (distribution of data given the tree structure) over all accepted regression tees are displayed in Table 4. The Bayesian regression tree algorithm search space includes trees with low to high RSS, deviance and log posterior. There was no evidence over-fitting with Bayesian regression trees since there was little difference in log RSS and deviance between training and validation datasets.

**Table 4:** Quantiles of log residual sums of squares (RSS), deviance and log posterior for training and validation datasets over all accepted trees, for Bayesian regression trees.

		2.50%	50%	97.50%
Training	Log RSS	−55.446	−51.261	−49.960
	Deviance	10.213	21.224	40.428
	Log posterior	−21.284	−12.823	−12.478
Validation	RSS	−61.689	−57.727	−55.864
	Deviance	8.597	14.823	28.864
	Log posterior	−17.232	−10.846	−9.879

### Spatial CAR model

Table 5 shows that under the spatial regression (CAR) model, the average increase in monthly cryptosporidiosis incidence rates was 9% (95% credible interval (CrI): 0–18%) for a 1°C increase in monthly average maximum temperature. However, there was no substantive association between SEIFA, rainfall and cryptosporidiosis incidence. No interactions effects were found between temperature and SEIFA.

**Table 5:** Changes (%) in relative risks with 95% credible intervals from Bayesian spatiotemporal CAR models of cryptosporidiosis in Queensland, Australia.

Variables	Posterior mean	SD	MC error	RR (95%CI)
**Temperature (°C)**	0.1046	0.0440	<0.01	1.11 (1.02–1.21)
**SEIFA**	−0.0003	0.0025	<0.01	1.00 (0.99–1.01)
**Rainfall (mm)**	0.0003	0.0009	<0.01	1.00 (0.99–1.01)
**Temperature×SEIFA**	0.0005	0.0004	<0.01	1.00(0.99–1.01)

### Comparison with frequentist CART models

We also compared the outcomes of the Bayesian CART model with those of the traditional CART model [8]. Both the Bayesian CART and traditional CART models show that SEIFA and temperature were associated with the cryptosporidiosis disease. However, the analyses indicate that Bayesian CART gave slightly better prediction accuracy (ie. high sensitivity) (sensitivity_Bayeisan:_79%; specificity_Bayesian:_ 50%) than the CART accuracy (sensitivity_frequentist:_ 10%; specificity_frequentist:_ 99%) established using the more traditional frequentist approach. An important difference between the two models was that the frequentist tree gave equal weighting to correct classification of all observations, whereas the Bayesian tree differentially weighted the groups of presences and absences based on the respective sample size.

## Discussion

Both the Bayesian CART and Bayesian CAR models show that temperature was significantly associated with the cryptosporidiosis disease. The analyses indicate that the nature and magnitude of the effect estimates were similar for the two methods used in this study. However, the Bayesian CART allowed more flexible identification and description of nonlinear interactions between explanatory or predictor variables, while still allowing for local smoothing.

The Bayesian CART model revealed a strong nonlinear interaction between SEIFA and temperature, and a weaker interaction with rainfall, in predicting incidence rate of cryptosporidiosis. In contrast, because only main effect term and one interaction term (ie. temperature and SEIFA) were included in the spatial CAR model, other interactions were not identified. Although other interactions (ie. temperature, rainfall and SEIFA) could of course be included in the CAR model, it is difficult to identify *a priori* which interactions to include and evaluation of all possible interactions would require a much larger dataset than was available here.

We also considered including these interactions in a spatial CAR hurdle model, which allows for zero-inflation by having a probability mass at zero, but found this to be difficult to fit in terms of stability and interpretability of the estimates and corresponding predictions. This is possibly not surprising given that the discretisation of the data into two components (zero and non-zero) may impact on the representation of the spatial component in the model, especially when taking into interactions into account. This requires further future investigation. In the meantime, *a posteriori* inclusion of interactions, based on the CART, into the CAR model analyses is a potentially useful alternative.

A strong advantage of a Bayesian framework for the CAR and CART models is that all the parameters of the model are treated as variables, so that probabilistic inferences are made on the basis of the corresponding posterior distributions [30]. Moreover, by virtue of the MCMC computation, the distributions used to describe these variables are no longer constrained to analytically tractable (e.g., normal) formulations. Furthermore, under a Bayesian CART framework, a diverse range of tree structures can be readily explored. The typical frequentist approach of fitting the CART model uses single recursive partitioning algorithms [31], [32] in which the choices of the splitting rules at nodes further down the tree are constrained by the choices made at nodes above it, and only get one optimal tree. In contrast, the Bayesian CART approach investigates a wide variety of tree structures with different variables, splitting rules and number of terminal nodes. At any splitting node, the variable and splitting rules are randomly selected from the prior and trees that perform well in terms of high likelihood (low deviance) and posterior probabilities are chosen. Accounting for model uncertainty in this manner can improve predictive performance [8].

A Bayesian CART model for identification and estimation of the spatial distribution of disease risk can be useful in monitoring and assessment of infectious diseases and in decision-making about prevention and control. The methodology developed through this study may be directly applicable to research on other infectious diseases, with further potential for application to a wider range of public health problems.
